# Design of Complexly Graded Structures inside Three-Dimensional Surface Models by Assigning Volumetric Structures

**DOI:** 10.1155/2019/6074272

**Published:** 2019-02-05

**Authors:** Ronny Brünler, Robert Hausmann, Maximilian von Münchow, Dilbar Aibibu, Chokri Cherif

**Affiliations:** Institute of Textile Machinery and High Performance Material Technology (ITM), Technische Universität Dresden, Hohe Str. 6, 01069 Dresden, Germany

## Abstract

An innovative approach for designing complex structures from STL-datasets based on novel software for assigning volumetric data to surface models is reported. The software allows realizing unique complex structures using additive manufacturing technologies. Geometric data as obtained from imaging methods, computer-aided design, or reverse engineering that exist only in the form of surface data are converted into volumetric elements (voxels). Arbitrary machine data can be assigned to each voxel and thereby enable implementing different materials, material morphologies, colors, porosities, etc. within given geometries. The software features an easy-to-use graphical user interface and allows simple implementation of machine data libraries. To highlight the potential of the modular designed software, an extrusion-based process as well as a two-tier additive manufacturing approach for short fibers and binder process are combined to generate three-dimensional components with complex grading on the material and structural level from STL files.

## 1. Introduction

Additive manufacturing technologies are based on the layered construction of material into a finished component. The production is carried out by solidification of

- Liquids or gels (curing, drying, crosslinking, etc.) [1–9]
- Powders or granules (gluing, sintering, etc.) [10–16] or
- Pasty and belt-shaped or strand-shaped materials (direct deposition without curing processes or solidification, etc.) [17–20]

The limitations for realizing parts with a complex material composition are either found in the process characteristics or in the data formats used.

Technologies using powder beds or liquid baths are limited to a particular material or a particular material composition which is constant throughout the entire component [21, 22]. With these methods, different materials cannot be applied within a part, in particular not within one layer.

In the case of the open-space or layer construction methods, it is possible to use various materials [23, 24].

However, the limitations for parts with complex material and structural composition lie in the properties of the file formats [25]. The most common file format in generative production methods is the STL format (standard tessellation language/standard triangulation language) [26–28]. In niche applications, the AMF format (additive manufacturing format) and the OBJ format (object format) are used as well [29, 30].

**Figure 1:**
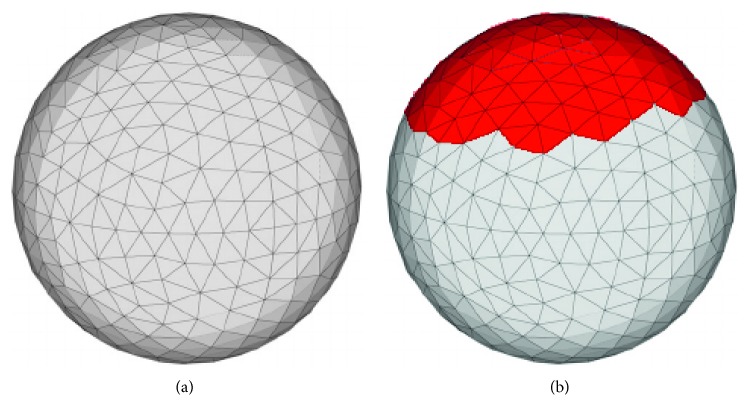
(a) Visualization of the surface representation of a sphere by means of triangles in the STL format [31]. (b) The same sphere in the AMF or OBJ format that allow assigning descriptive metadata to individual triangles, shown here as red color scheme in the upper part of the sphere.

A sphere is used to illustrate the file formats. The STL file format provides only surface information and uses triangles, often referred to as vertices, to represent the surfaces as shown in Figure 1(a). In the AMF or OBJ format, the surface information can also be supplemented with properties (material, textures, or metadata), emphasized by a red color scheme in the triangles on the top of the sphere in Figure 1(b).

**Figure 2:**
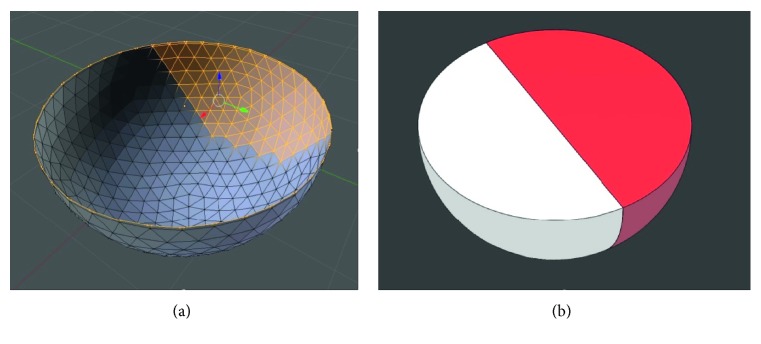
(a) Visualization of the structure of the sphere in surface-based formats that solely allow selecting and thus altering the surface triangles. (b) Image of a sphere with two colors designed in a CAD software as a solid body.

STL files as well as AMF or OBJ files represent geometric bodies exclusively by means of surface information. Regardless of whether the files are stored as surface or solid bodies, they contain no volume data. Basically, the objects are hollow inside and have “outer walls” that are infinitesimally thin. The only difference in solid bodies is the representation of a filled body.  Figure 2(a) shows a graphic representation of the previously discussed sphere cut in half using the software Blender (Blender Foundation). As in all other software for editing or displaying STL files or similar formats (Netfabb, Cura, Slic3r, and Repetier, amongst others) as well as CAD software (FreeCAD, SolidWorks, AutoCAD, and CATIA, amongst others), only the surface of the structure can be addressed as it is simply impossible to select or click on other structures than the surface triangles. This points up the decisive limitation of all surface-based file formats: within surface-approximated geometries, for example, from computer tomography recordings (CT) and magnetic resonance tomography representations (MRT) or 3D scans, property assignments cannot be implemented.

In CAD programs, however, objects that contain volume information can be designed and stored in the AMF or OBJ format, but again have to be regarded as individual surfaces approximated by triangles. It is thus possible to realize structures with grading on the material or structural level, supposing that they are designed from scratch as the two-color sphere in Figure 2(b).

**Figure 3:**
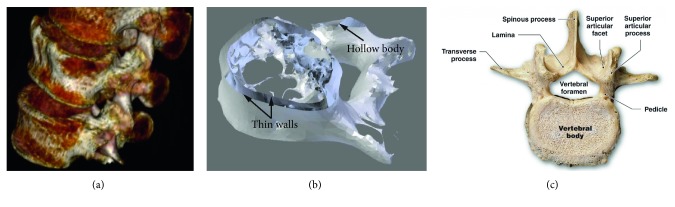
(a) CT scan of the lower spine showing the L3 (top), L4 (middle), and L5 (bottom) lumbar vertebrae [32]. (b) View of the STL file of an L5 lumbar vertebra from [32] exhibiting infinitesimally thin walls and a hollow body. (c) Anatomy of the lumbar vertebrae [33].

However, assigning properties within previously defined or given bodies as obtained from CT scans, MRI scans, or radiographs for the determination of defect geometries in regenerative medicine or 3D scans from reverse engineering cannot be carried out in these programs because of the surface-based representation and the related restrictions.  Figure 3(a) shows a CT scan of the lower spine and an STL file derived from that scan containing geometry information of a lumbar vertebra exhibiting a complex geometry. Substantial differences in the structural composition of the STL file can be observed in comparison with the anatomy of a vertebra (Figure 3(c)). The STL file features a hollow body and shows almost no structures in the area of the spongy bone in the center of vertebral body and infinitesimally thin walls instead of the dense cortical bone around the spongy bone structure. There is no software yet available to fill certain regions with different materials or structural variations within STL, AMF, or OBJ files of parts with complex geometry.

## 2. Materials and Methods

### 2.1. Software for Accessing the Inner Structure of Surface-Based Bodies

**Figure 4:**
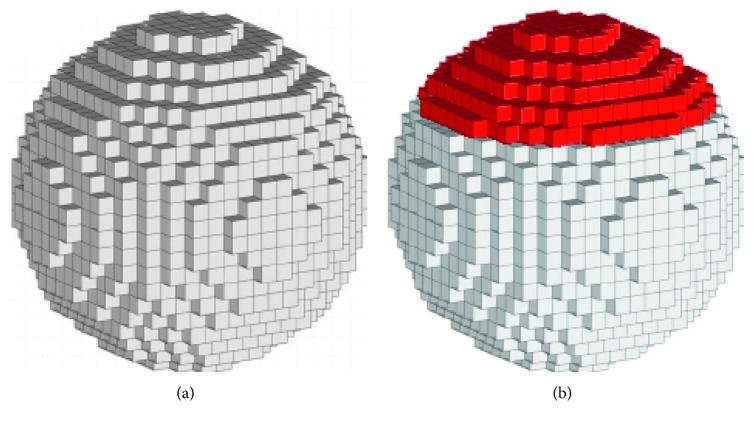
(a) Visualization of a sphere in voxel format. (b) The same sphere with a red color scheme in the upper part of the sphere. Descriptive metadata can be assigned to individual voxels even throughout the inner parts of the body.

In addition to surface-based file formats, bodies can also be represented by volumetric elements (voxels). Graphic representations of spheres in voxel format with assigned metadata are shown in Figure 4. This approach is mostly known from video games such as Minecraft (Mojang/Microsoft Studios, 2009) and Blade Runner (Virgin Interactive, 1997) or simulations [34] to represent terrain features and is also widely used in medical imaging formats such as DICOM® [35, 36]. However, the voxel formats are not used for designing implants for regenerative medicine, prosthetic components, or 3D printing applications in reverse engineering as slicer software is usually developed for STL files. Hence, while it is possible to 3D print or display complex geometries with different surfaces, it is not possible to realize grading on the material or structural level inside the structures.

**Figure 5:**
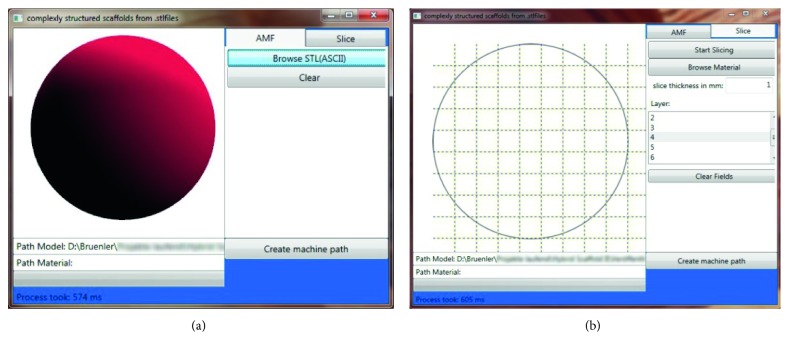
Graphical user interface of the developed software: (a) “AMF” tab with imported sphere; (b) “Slice” tab showing the midlayer of the subdivided sphere, the input field for adjusting slicing thickness, and the scroll bar for scrolling through the single slices.

To access the inner structure of surface-based bodies, novel software for segmenting the structures into voxels and manipulating them is developed. The software is capable of processing STL files in ASCII format and is developed in C# within the development environment Visual Studio Community (Microsoft Corp.).  Figure 5 shows the graphical user interface (GUI) that was created within the Windows Presentation Foundation (WPF) framework.

After importing a file, it is automatically converted into the AMF format and can be viewed, rotated, and zoomed in and out on the “AMF” tab (Figure 5(a)). The “Slice” tab as visualized in Figure 5(b) is used to slice the body and to additionally implement a rectangular grid. Slicing thickness and grid size can be adjusted arbitrarily and separately. In default mode, the grid size matches the slicing thickness. Thus, the grid subdivides the body into cubic voxels.

**Figure 6:**
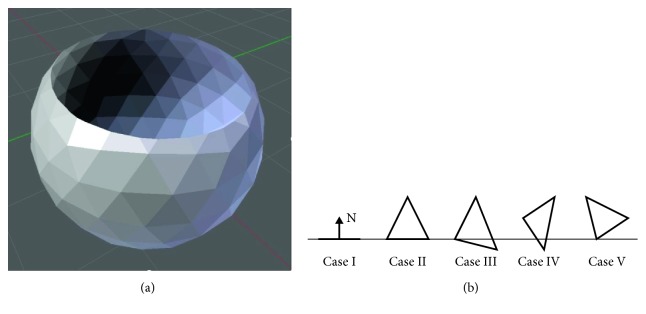
(a) Slicing of a sphere with resulting cuts of the surface triangles. (b) Specific cases for situational relations between the section plane and surface triangles during slicing [37].

Generating the slice data for a graphical representation of the contours of the body requires the consideration of several special cases related to the surface representation by means of triangles. During the generation of a single slice, necessarily some triangles are cut by the section plane as evident in Figure 6(a). In total, there are 10 cases describing the situational relations between section plane and triangles [37]. While simple cases such as triangles located completely above or below the section plane are easy to process, five specific cases have to be considered more closely (Figure 6(b)):

1. All three edges are located on the section plane
2. Exactly two edges are located on the section plane
3. One edge is located above, one below, and one on the section plane
4. One edge is located above/below the section plane and two edges are located on the other side
5. Exactly one edge is located on the section plane

**Figure 7:**
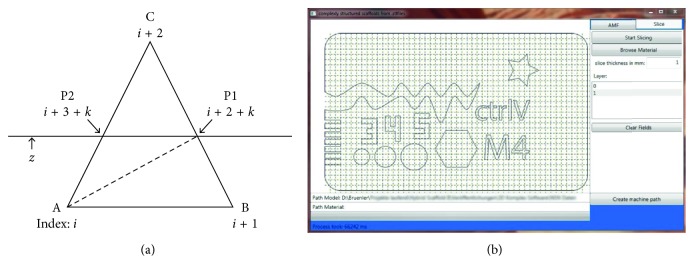
(a) Method for calculating the polygon course for a graphic representation of single slices. (b) Polygon course of the base layer of a complex 3D structure [38].

These considered cases have a significant effect on the calculation of the intersection points. The coordinates of the edges of all triangles describing the edited body are stored in a separate class (triangle class) within the software. They are processed during slice data generation and serve for intersection point calculation. After using a certain coordinate for calculation, it may be erased from the to-be-processed data. However, depending on the case and on the ratio between slicing thickness and triangle size, certain coordinates have to be used for the calculation of the next layer and must not be erased. To ensure a correct calculation, novel triangles are generated according to Figure 7(a). The triangle (defined by a, b, and c in Figure 7(a)) he is divided into three smaller triangles using the intersection points (P1 and P2) with the current section plane. In the case described here, the coordinates a and b can be erased from the triangle class, and only the triangle defined by P1, P2 and C is used for calculating the intersection points in the next layer.

After calculation of all intersection points, a polygon course is generated automatically and displayed for each layer in the slice tab. The software layout also allows processing more complex bodies, such as the “test your 3D printer! v2” file from Thingiverse (MakerBot Industries, LLC) as illustrated in Figure 7(b) [38].

## 3. Results and Discussion

### 3.1. Assignment of Multiple Properties and g-Code to Subvolumes within Given Complex Geometries

The imported body is divided into voxels by the rectangular grid implemented in the “Slice” tab. Thus, all voxels are accessible by scrolling through the single layers. These features enable assigning arbitrary properties to every single voxel within any given geometry.

**Figure 8:**
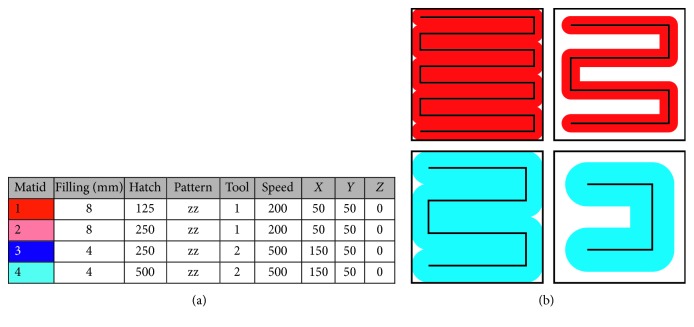
(a) Standard file for extrusion-based printing with two different colored materials and different deposition patterns. The voxel size in this figure is 1 mm and is filled in *z* direction with 8 red material strands (tool 1; red material; strand thickness 125 *µ*m; traversing speed 200 mm/min; zero position of the nozzle with respect to tool installation in our machine) and 4 blue strands (tool 2; blue material; strand thickness 250 *µ*m; traversing speed 500 mm/min; zero position of tool #2), leading to (b) different voxel filling patterns with adjusted path distances (hatch) for the same material used.

The properties assigned to each voxel are used to generate machine-readable code. As g-code is the most common numerical control programming language, it is used for further processing. Specific commands are filed by means of a user-friendly editable .txt file. It can be adjusted depending on the manufacturing technology used. The standard file for extrusion-based additive manufacturing processes is shown in Figure 8(a). It contains the material id (matid), the number of layers necessary to fill one millimeter (fillings/mm), the path distance (hatch), the path arrangement (pattern), the digital output command being used to control the extrusion nozzle (tool), the speed of the tool (speed), and the zero position of the tool used (*X*-, *Y*-, and *Z*-coordinates). The strand thickness can be calculated from the “fillings/mm” column as these values are used for the travel of the *z*-position to lay the strands directly on top of each other. Thus, according to Figure 8(a), 8 fillings/mm are used for a strand thickness of 125 *µ*m and 4 fillings/mm are used for 250 *µ*m strands, respectively.

The absolute positions of the voxels and the assigned fillings, hatches, tools, patterns, speeds, and positions are used to generate g-code commands. The black paths in Figure 8(b) directly show the course of the generated path in g-code. Travels in *z* direction are handed over from the information provided by the “fillings/mm” column and the slice thickness.

The file shown in Figure 8(a) leads to different material deposition. The patterns with “matid 1” and “matid 2” are extruded from the same nozzle (tool 1) and with the same speed but in different path distances. For “matid 1,” the strand thickness matches the path distance and thus fills the voxel area in top view creating a dense structure (Figure 8(b), top left). “Matid 2” deposits the 125 *µ*m strands in a path distance of 250 *µ*m and thus exhibit filling levels of 50%. In “matid 3” and “matid 4,” another tool is used to deposit larger strands in different patterns.

**Figure 9:**
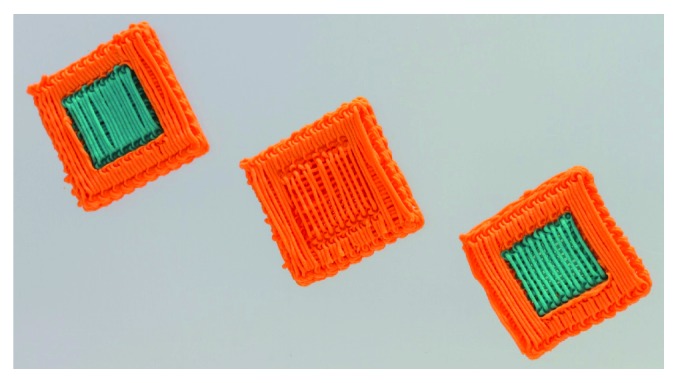
Manufacturing of material grading (left), porosity grading (center), and material and porosity grading (right) by means of assigning different nozzles (tool column) and strand spacings (hatch column).

Figure 9 shows three different structures manufactured from the same cuboid STL file (20 mm × 20 mm × 4 mm). Two extruding systems with nozzles 0.4 mm in diameter (Nordson EFD) filled with different colored clay were used to manufacture different structures within the STL file.

The left structure was manufactured with both nozzles using the same path distance leading to a laydown of one material (light-blue colored clay) in the inner zone and the other material (orange colored clay) in the outer part while both regions featuring a dense path spacing. The structure in the middle was manufactured using solely one nozzle following dense path spacing in the outer part and a 0.8 mm path spacing in the inner region leading to 50% porosity. The structure on the right was manufactured with both nozzles using different path distances generating a structure featuring both different materials and different path spacing and thus porosity. The different material grading, porosity grading, or combination of both within the lattice structures were realized within a STL file that usually solely defines the outer geometry by making use of two extrusion nozzles.

### 3.2. Additive Manufacturing of Complexly Structured Lattice Structures from Surface Models

The software is designed to assign the information from the editable configuration file to the voxels generated by slicing and gridding of the imported surface based file. An easy-to-use graphical user interface was developed to display the voxels and the boundary curve(s) of the imported body as well as the basic properties of the material deposition as defined in the editable .txt file.

**Figure 10:**
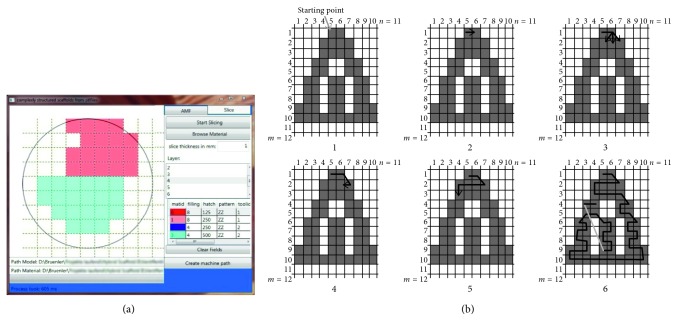
(a) “Slice” tab showing assigned structures from the browsed standard file within the midlayer of a 9 mm sphere. (b) Zig-zag algorithm to create continuous paths in complex slice geometries according to [39].

Figure 10(a) shows the GUI of the software during assigning materials from the standard file to one layer of the previously discussed sphere. The standard file is displayed in a reduced form to provide a large area for assigning the materials and patterns.

**Figure 11:**
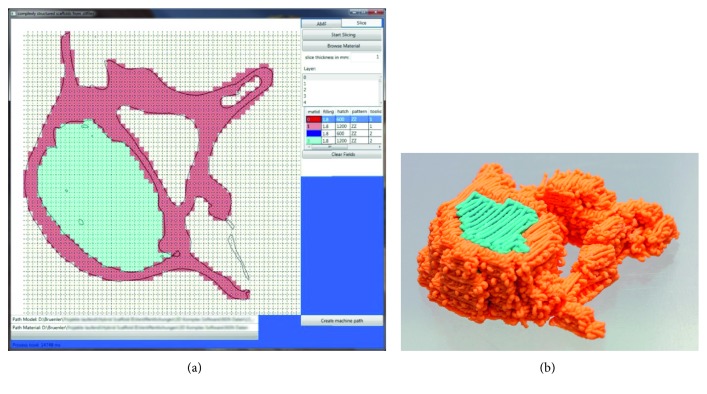
(a) GUI of the software with a large area (left) for assigning the voxel parameters from the standard file (right) with polylines representing the section plane and a field for scrolling through the layers (upper right). (b) Printed body featuring different colors as well as different strand spacing to demonstrate the software's ability to generate different g-codes for different nozzles based on the part geometry and the user's assignments.

The voxel size and overall structure size are not limited by the software. However, the voxel size should be dimensioned according to the tools used. In the case of extrusion-based processes, the strand width and the strand spacing should be considered. An appropriate voxel size for the extrusion nozzles used for manufacturing the structures from Figure 9 may lead to different relative disparities in geometries with different sizes. The comparison between Figures 10(a) and 11(a) shows larger relative deviations in the sphere with 9 mm in diameter and good conformity for the life-size vertebra while using the same voxel size. The processing time is dependent on the number of surface triangles of the processed structure and the hardware used. The process of loading and processing the vertebra structure that is defined by 34464 surface triangles into voxels took 71.6 seconds on a dual-core 2.4 GHz, 4 GB RAM, 256 MB graphics memory system and 14.5 seconds on an eight-core 3.4 GHz, 32 GB RAM, 1 GB graphics memory system, making the software applicable on a wide range of computing systems. Contiguous areas are processed with continuous paths by using the zig-zag algorithm according to Figure 10(b).

To show the potential of the novel developed software, the surface model of a human lumbar vertebra, extracted from a CT scan and provided as STL file by MarioDiniz on Thingiverse [32] as shown in Figure 3, is subdivided in voxels and filled with different materials and patterns. The software GUI in Figure 11(a) shows polylines based on the calculations presented, showing walls, remains from scanning parts of the trabecular bones, and artefacts within the STL file. These lines function as guidance for assigning the materials and structures from the editable configuration file to the part. For manufacturing the part, two extrusion-based nozzles with a diameter of 0.4 mm (Nordson EFD) as in Figure 9 are used. The example part features an orange pattern with narrow strand spacing of 400 *µ*m for the areas of the cortical bone (compact bone) and a light-blue pattern with a strand spacing of 800 *µ*m, leading to a porosity of about 50% in the areas of the cancellous bone (spongy bone) of the vertebral body.  Figure 11(b) shows the printed body featuring a material grading (different colors) as well as a porosity grading (strand spacing).

### 3.3. Combined Additive Manufacturing of Complex Fiber-Based and Strand-Based Structures for Biomedical Applications

The applications shown so far relate exclusively to the production of extrusion-based structures. The adaptation of the configuration file is used directly to automatically create a g-code with corresponding strand spacing. The software, which also serves as a postprocessor, also allows the use of completely different additive manufacturing processes.

The Net Shape Nonwoven Method (NSN) is a unique technology for the additive production of short fiber-based structures for regenerative medicine [40]. Similar to powder bed printing, this technology is a two-tier process. First, a thin fiber bed is applied, and subsequently a binder is applied to selectively bonding the fibers together. Using the customizable configuration file, the developed modular software also allows the production of NSN structures. By appropriate selection of the tools for fiber application units (actuated by speed-controlled stepper motors) and the path distances, either a full surface fiber application or a local fiber application can be realized. Clearly, the width of the fiber track depends on the fiber length used and usually ranges from 0.5 mm to 2 mm. With these fiber lengths, which can be estimated simulation-based, suitable pore sizes and porosities for regenerative medicine can be generated [41]. For the second process step, a piezo-controlled adhesive nozzle is actuated. In order to achieve precise contours and geometries, path distances of about 200 *µ*m are used.

**Figure 12:**
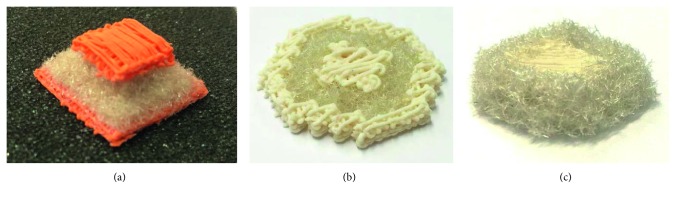
Unique structures combining extrusion-based additive manufacturing processes with a fiber-based additive manufacturing approach. (a) Sandwich structure, (b) core-shell-shell structure, and (c) core-shell structure, demonstrating uses in different applications.

Due to the flexible controlling of extrusion nozzles, fiber application units, and adhesive nozzles, completely different additive manufacturing processes can be combined to create novel structures.  Figure 12 shows different structures on the basis of simple STL files into which different materials have been inscribed.

The newly developed approach allows assigning volumetric structures in three-dimensional surface models. STL files obtained from CT or MRI scans in medicine, 3D scans from reverse engineering, CAD software, or any other sources solely contain information of the surface of the bodies. With existing software, assigning properties within the bodies is not possible as only triangles on the surface area can be selected.

The hosted editable configuration file allows controlling different extrusion nozzles, fiber application units, and a piezo-driven adhesive nozzle (via tool column). It features arbitrary nozzle diameters, leading to different strand thickness or different fiber layer heights (via filling/mm column). All materials are deposited in a z-pattern (via pattern column) starting in the *x* direction in the first layer of a voxel and subsequently changing its direction into the *y* direction which ensure a crossing of the strands and thus structural stability especially for strand deposition. The standard file also allows setting the speed (in mm/min via the speed column) and adjusting the tool position in the machine (in absolute *XYZ* coordinates (mm) in the respective columns). The “matid” column shows a colored pattern with dark and light colors representing the materials as well as the porosity to simplify the material/pattern choice during assigning the properties from the .txt file to the body.

After assigning the parameters to the voxels, a g-code can be generated automatically by clicking the “Create machine path” button on the lower right (see Figure 11(a)). Machine paths of adjacent voxels featuring the same “matid” are handed over to g-code as a continuous line and are processed according to the zig-zag pattern shown in Figure 10(b).

The possible use of the software goes beyond the usage as a postprocessor for extrusion-based or fiber-based additive manufacturing as it basically allows assigning any control commands to every single voxel:

- Positioning and travel commands for axes (e.g., XY tables and XYZ tables)
- On/off and/or speed commands for motors (e.g., material feed/deposition/compaction/removal and applying of substrates)
- Setting/resetting of digital or analog outputs (e.g., connected extruders, nozzles, heaters, coolers, fans, and lasers)
- Transfer information to other units (e.g., bus systems, direct digital controls, robot controls, displays, and user feedback)

Thus, any processing technology or application may be implemented by customizing the spreadsheet file or implementing other (machine-readable) codes according to the users' needs.

Furthermore, the voxel-based approach allows assigning any information (e.g., materials, material morphologies, colors, porosities, and metadata) to the imported files. The information may be stored and used for other applications or further processing.

## 4. Conclusions

The presented method allows slicing and gridding bodies from STL or AMF files into volumetric elements (voxels) of arbitrary size. The underlying software allows assigning different tools and features such as path distances, strand thicknesses, or traveling speeds to each of these voxels. Adjoining voxels with equal properties are combined to subvolumes and may either be manufactured into structures with material grading, porosity grading as well as combinations of both within the different regions or be stored in AMF format and be used in compatible software or printing technologies.

Strand-based lattice structures with grading on the material and structural level can be designed and manufactured within a multinozzle additive manufacturing approach and can furthermore be combined with a two-tier process for fiber-based additive manufacturing well suited for applications in regenerative medicine. The combination of both approaches enables, e.g., designing press-fit applications with flexible transition areas on the basis of geometry data from complex defects. Furthermore, large defects affecting different tissue types or tissue morphologies, e.g., osteochondral defects involving bone and cartilage, may be addressed.

## Data Availability

The software used to support the findings of this study is described extensively within the article. The described findings can be used to replicate the findings of the study. Furthermore, parts of the software used to support the findings of this study are available from the corresponding author upon request.
